# First person – Hiroto Yamamoto

**DOI:** 10.1242/bio.062785

**Published:** 2026-07-28

## Abstract

First Person is a series of interviews with the first authors of a selection of papers published in Biology Open, helping researchers promote themselves alongside their papers. Hiroto Yamamoto is first author on ‘ Three-dimensional analysis of spawning behaviour of skipjack tuna *Katsuwonus pelamis* using stereo-image measurements’, published in BiO. Hiroto conducted the research described in this article while at Yasushi Mitsunaga and Shinsuke Torisawa's lab at the Graduate School of Agriculture, Kindai University, Nara, Japan. He is now a PhD student in the lab of Takashi Kitagawa at The University of Tokyo, Japan, investigating the adaptive significance of animal behaviour and ecology.

**Figure:**
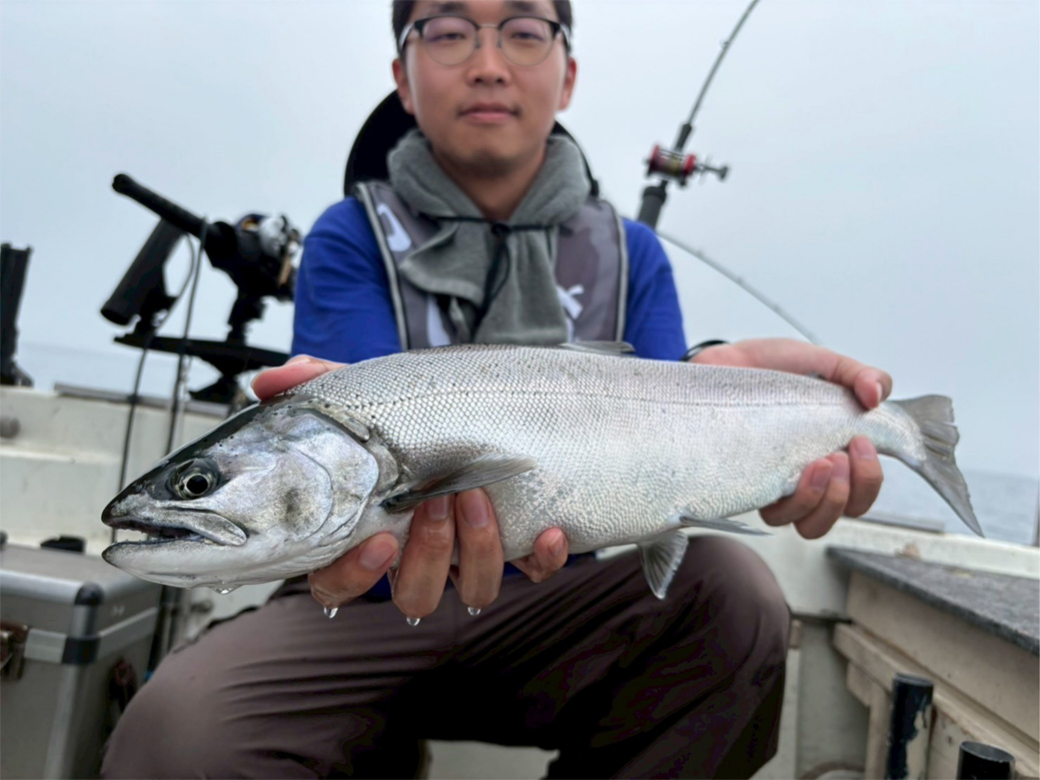
Hiroto Yamamoto

**Describe your scientific journey and your current research focus**

My scientific journey began as an undergraduate, when I joined a research project at a national research institute as a research assistant. The project, conducted at Kagoshima City Aquarium, investigated how water temperature affects the hatching rate and time of skipjack tuna eggs. During the study, I witnessed skipjack tuna spawning for the first time and became fascinated by their reproductive behaviour. That experience inspired me to pursue my Master's research on the species, which led to the study published in this journal. Through my work on skipjack tuna, I developed a broader interest in the ecology of migratory fishes and joined my current supervisor's laboratory. My current research focuses on the migratory ecology of a salmonid fish inhabiting Lake Biwa, Japan.

**Who or what inspired you to become a scientist?**

The researchers and aquarium staff I worked with during my first research experience as an undergraduate were my greatest inspiration. Their enthusiasm for science and the opportunity to work alongside them showed me how exciting research could be. Without that experience, I do not think I would have chosen to pursue a career in science.

**How would you explain the main finding of your paper?**

Although scientists have learned a great deal about how male and female fish behave during reproduction, surprisingly little is known about species that gather in large groups to spawn in the open ocean. In this study, we made the first detailed observations of the spawning behaviour of skipjack tuna. By analysing underwater videos, we were also able to estimate the size of the fish and how fast they were swimming. We found that most spawning events involved just one presumed male and one presumed female, while events involving several fish were uncommon. During these events, the leading fish sometimes swam at speeds of up to nine times its own body length per second. If the leading fish is the female, as we suspect, this remarkable speed may play an important role in successful reproduction.

…combining behavioural observations with individual identification and genetic parentage analyses could reveal how males and females maximize their reproductive success

**Figure:**
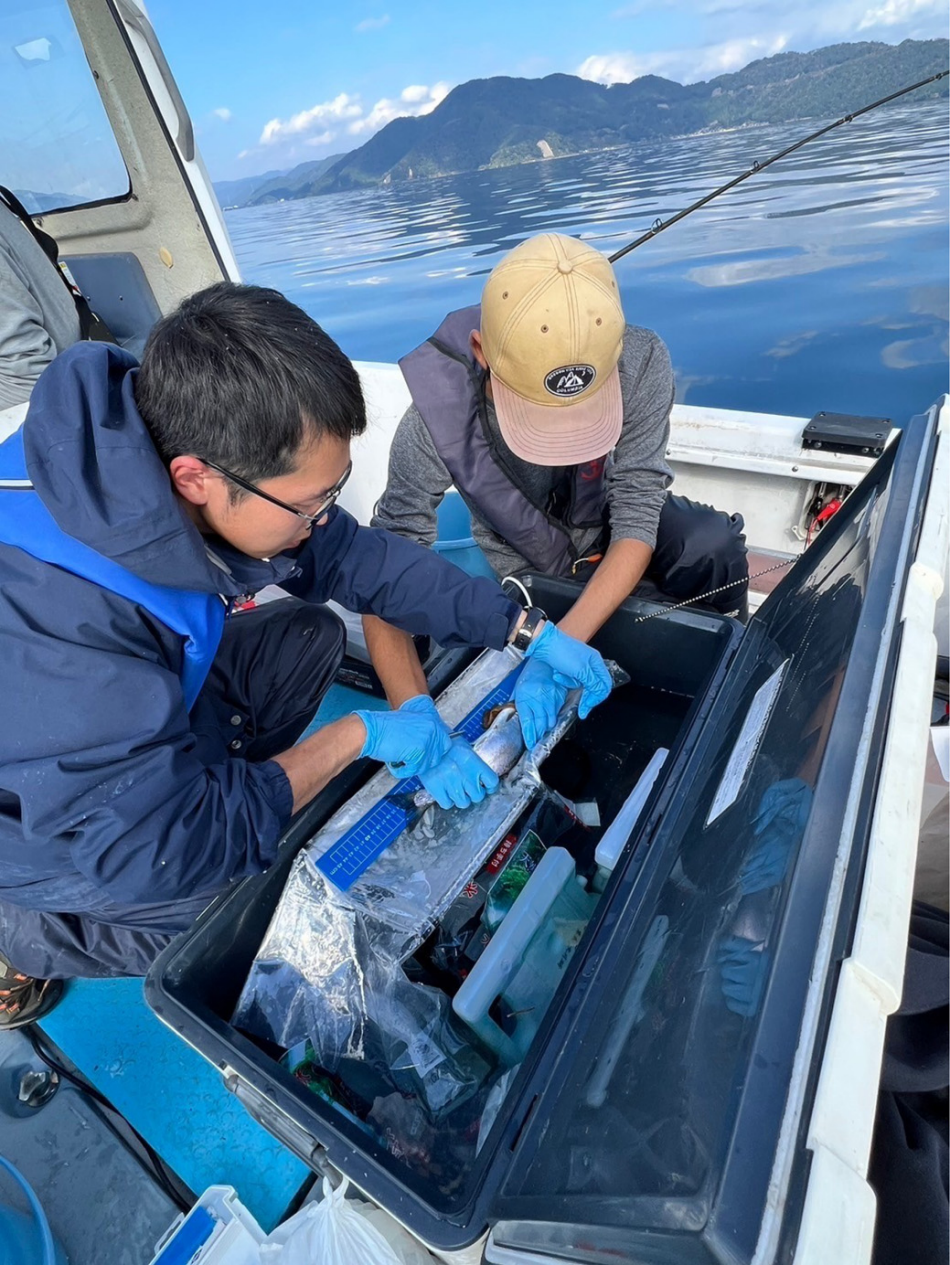
**Field work in Lake Biwa, Japan.** We attached archival tags and ultrasonic transmitters to Biwa salmon (*Oncorhynchus biwaensis*).

**What are the potential implications of this finding for your field of research?**

Because spawning in large groups is difficult to observe in the open ocean, it has remained one of the least understood aspects of the reproductive biology of pelagic fishes. Our study provides one of the first detailed descriptions of this behaviour in skipjack tuna and offers a foundation for future research. Although we were not able to determine the reproductive strategies underlying these spawning events, combining behavioural observations with individual identification and genetic parentage analyses could reveal how males and females maximize their reproductive success. I hope our findings encourage further studies of spawning behaviour in other pelagic fish species.

**Which part of this research project was the most rewarding?**

The most rewarding part of this project was being able to study the spawning behaviour of skipjack tuna, which had first fascinated me as an undergraduate. What began as simple curiosity grew into a research project that allowed me to analyse this remarkable behaviour in detail. It was incredibly rewarding to present the findings at an international symposium and, ultimately, to publish the work in this journal. Seeing a question that first sparked my interest develop into a published study has been a truly fulfilling experience.

**What's next for you?**

I am currently a PhD student studying the migratory ecology of a salmonid fish in Lake Biwa, Japan, and my immediate goal is to complete my doctorate. After that, I hope to continue a career in research, exploring the questions about animal behaviour and ecology that I find most fascinating. Curiosity has guided my scientific journey so far, and I hope it will continue to do so in the future.
